# Anti-pancreatic cancer activity of ONC212 involves the unfolded protein response (UPR) and is reduced by IGF1-R and GRP78/BIP

**DOI:** 10.18632/oncotarget.20819

**Published:** 2017-09-12

**Authors:** Avital Lev, Amriti R. Lulla, Jessica Wagner, Marie D. Ralff, Joshua B. Kiehl, Yan Zhou, Cyril H. Benes, Varun V. Prabhu, Wolfgang Oster, Igor Astsaturov, David T. Dicker, Wafik S. El-Deiry

**Affiliations:** ^1^ Department of Hematology/Oncology, Laboratory of Translational Oncology and Experimental Cancer Therapeutics, Molecular Therapeutics Program, Fox Chase Cancer Center, Philadelphia, PA, USA; ^2^ Biostatistics Department, Fox Chase Cancer Center, Philadelphia, PA, USA; ^3^ Massachusetts General Hospital, Boston, MA, USA; ^4^ Oncoceutics, Inc., Philadelphia, PA, USA; ^5^ Department of Hematology/Oncology, Molecular Therapeutics Program, Fox Chase Cancer Center, Philadelphia, PA, USA

**Keywords:** pancreatic cancer, ONC201, ONC212, AG1024, IGF1-R

## Abstract

Pancreatic cancer is chemo-resistant and metastasizes early with an overall five-year survival of ∼8.2%. First-in-class imipridone ONC201 is a small molecule in clinical trials with anti-cancer activity. ONC212, a fluorinated-ONC201 analogue, shows preclinical efficacy in melanoma and hepatocellular-cancer models. We investigated efficacy of ONC201 and ONC212 against pancreatic cancer cell lines (*N*=16 including 9 PDX-cell lines). We demonstrate ONC212 efficacy in 4 *in-vivo* models including ONC201-resistant tumors. ONC212 is active in pancreatic cancer as single agent or in combination with 5-fluorouracil, irinotecan, oxaliplatin or RTK inhibitor crizotinib. Based on upregulation of pro-survival IGF1-R in some tumors, we found an active combination of ONC212 with inhibitor AG1024, including *in vivo*. We show a rationale for targeting pancreatic cancer using ONC212 combined with targeting the unfolded-protein response and ER chaperones such as GRP78/BIP. Our results lay the foundation to test imipridones, anti-cancer agents, in pancreatic cancer, that is refractory to most drugs.

## INTRODUCTION

Pancreatic cancer is a devastating disease with poor prognosis and an overall five-year survival rate of 8.2% (according to NCI Surveillance, Epidemiology and End Results (SEER) program) [1]. Surgery is the only cure, yet there are no effective screening methods that diagnose pancreatic cancer at the curative stage and less then 20% of diagnosed patients are eligible for surgery. Furthermore, the survival rate following surgery is only about 30%. In most cases, the primary tumor has already metastasized at the time of diagnosis [2, 3]. Current therapies available for pancreatic cancer patients include gemcitabine mono-therapy or combination therapies such as FOLFIRINOX, gemcitabine plus nab-paclitaxel, or gemcitabine plus erlotinib [4, 5]. In spite of the recent development of new chemotherapeutics, patients show poor response. In addition, although intensive research led to a better understanding of the biology of pancreatic cancer and to the identification of new targets for treatment; targeted therapy has shown limited success thus far. Targeted therapy for pancreatic cancer aims at targeting mutated oncogenes such as KRAS [6] or its downstream effector molecules like MEK/ERK [7], receptor tyrosine kinases including EGFR and IGFR [8, 9] and other molecules that are up-regulated in the tumor and promote survival and proliferation. Among the last group of molecules is the endoplasmic reticulum (ER) chaperone GRP78/BIP, a master regulator of the ER stress response known as a marker for poor prognosis in pancreatic cancer [10]. Therefore, there is an urgent need to develop novel targeted therapies for pancreatic cancer.

ONC201 is a first-in-class small molecule of the imipridone class of compounds. It demonstrates anti-tumor effects shown in a variety of cancer cell lines and in a panel of xenograft and orthotopic *in vivo* tumor models [11-22]. It is currently being tested in phase II clinical trials for solid tumors and hematological malignancies at several cancer centers. A first-in-human clinical trial in advanced solid tumors showed early signs of clinical benefit in advanced prostate and endometrial cancer patients [23]. In order to improve the efficacy of the parental compound, a group of ONC201 analogues were synthesized and initially tested on a number of cancer cell lines and xenograft models. In this screen, ONC212 showed improved efficacy in melanoma and hepatocellular carcinoma xenograft models [24]. Several studies indicate that ONC201 induces cellular stress response dependent on engagement of the ATF4 pathway [12, 25-27]. Similar to ONC201, ONC212 also induces the expression of CHOP, suggesting it is also inducing cellular stress. However, the mechanism of cellular stress following ONC212 treatment has not been fully elucidated [24].

In order to proliferate and activate pro-oncogenic signaling pathways, cancer cells upregulate different components of the UPR signaling pathway, such as constitutive activation of the IRE1α-XBP pathway or overexpression of GRP78/BIP [28]. This adaptive strategy increases the rate of protein synthesis and protein folding capacity of the ER, overall benefiting cancer cell survival. Altering the balance between the different components of UPR can affect cancer cell survival. Therefore, further induction of ER stress or targeting the UPR has been the goal in developing new drugs for cancer. Pancreatic cancer in particular is surrounded by a rigid stroma that induces hypoxic conditions. Hence, we hypothesized that ONC201 might have the potential to further induce ER stress in pancreatic cancer that will promote apoptosis. In addition, since pancreatic cancer exhibits resistance to many drugs and there is an immediate need for finding new therapies, we evaluated the new ONC201 analogue, ONC212, in pancreatic cancer. Consequently, the objective of this study was to determine the efficacy of ONC201 and ONC212 in pancreatic cancer as a single agent and potentially in combination with other drugs. We also aimed to elucidate the mechanism by which ONC201 and perhaps ONC212 induce cellular stress in pancreatic cancer.

## RESULTS

### Anti-proliferative effect of ONC212 is at least 10-fold more potent then ONC201 on a panel of 16 human pancreatic cancer lines (including 9 PDX cell lines)

**Figure 1:**
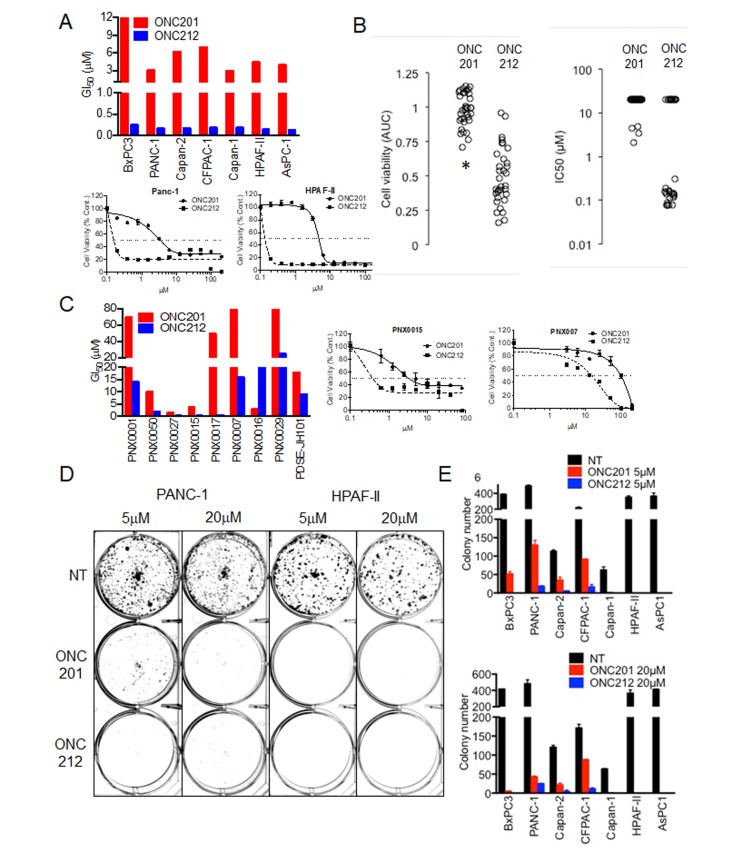
Anti-proliferative effect of imipridones ONC201 or ONC212 against human pancreatic cancer cell lines **A.** Effects on cell viability post ONC201 or ONC212 treatment was measured using CellTiter-Glo assay. (Top) Graphs representative of GI_50_ doses of ONC201 and ONC212 in a panel of seven human pancreatic cancer cell lines. (Bottom) representative dose-response curves of ONC201 and ONC212 in PANC-1 and HPAF-II pancreatic cancer cell lines. **B.** AUC and IC50 (µM) with 72 hour ONC201 and ONC212 (0.078-20 µM) treatment in a panel of 33 pancreatic cancer cell lines in the GDSC screen. * indicates *p* < 0.000005. **C.** (Left) Graphs representative of GI_50_ doses of ONC201 and ONC212 in nine low-passage patient-derived xenografted (PDX) pancreatic cancer cell lines. (Right) representative dose-response curves of two PDX lines. **D.** Colony formation assay post ONC201 or ONC212 treatment. Cells were treated with 5 µM or 20 µM of ONC201 or ONC212 for 72 hours and thereafter seeded in triplicate for each condition. Representative images of cells stained with crystal violet on Day 14 are shown. **E.** Colony number (*N* = 3) is represented graphically.

The anti-proliferative effect of ONC201 in comparison to ONC212 was first evaluated in a panel of seven pancreatic cancer cell lines and nine low-passage patient-derived xenografted pancreatic (PDX) cancer cell lines. Cell proliferation assay measured by CellTiter-Glo (CTG) revealed that at least a ten-fold lower concentration of ONC212 is needed to achieve 50% growth inhibition in comparison to ONC201. ONC212 showed GI_50_ values in the range of 0.1-0.4 μM, while the corresponding ONC201 GI_50_ values were in the range of 4-9 μM for the seven pancreatic cancer cell lines tested (Figure 1A, Supplementary Figure 1A and Supplementary Table 1). Significantly lower IC_50_ values of ONC212 compared to ONC201 were independently observed in a screen using the Genomic Drug Sensitivity in Cancer (GDSC) collection of pancreatic cancer cell lines (Figure 1B, and Supplementary Figure 1D). The low passage pancreatic cancer PDX cell lines exhibited 4-10 fold higher GI_50_ values for ONC201 compared to ONC212 (Figure 1B, Supplementary Figure 1A and Supplementary Table 1). Long-term cell proliferation assay showed that both ONC201 and ONC212 are comparable in inhibiting colony formation at a 20 µM dose. However, at a 5 µM dose, ONC212 was about 50-times more potent than ONC201 in preventing colony formation in four out of the seven pancreatic cancer cell lines tested (Figure 1C, 1D, and Supplementary Figure 1B). Similar differences in potency of ONC212 in comparison to ONC201 were observed by MTT assay (Supplementary Figure 1C). These results demonstrate the stronger anti-proliferative effect of ONC212 when compared with ONC201.

### ONC212 induces apoptosis earlier and at lower concentrations than ONC201 in sensitive pancreatic cancer cell lines

**Figure 2:**
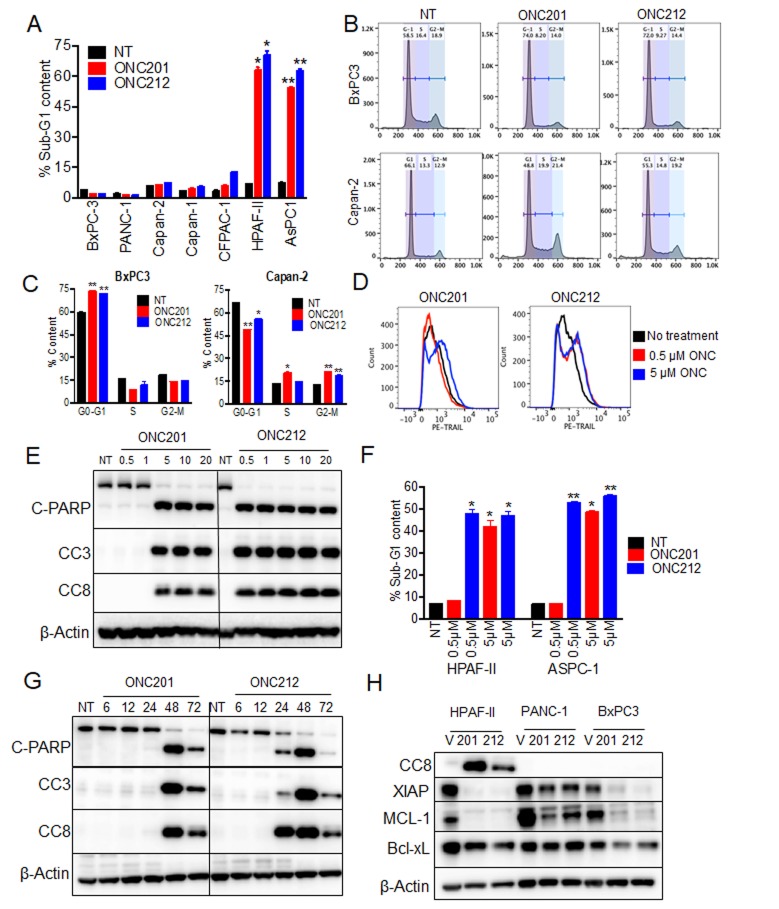
ONC212 induces apoptosis at lower doses and at earlier time point than ONC201 in sensitive cell lines **A.** Cell cycle profiles and percentage of apoptotic cells were evaluated in all seven pancreatic cancer cell lines post 20 µM ONC201 or ONC212 treatment. Changes in the sub-G1 phase of the cell cycle are graphically represented. **B.** Histograms of cell cycle analysis of BxPC3 and Capan-2. **C.** Quantification of cell cycle phases in BxPC3 and Capan-2 post 20 µM ONC201 or ONC212 treatment. **D.** Histograms representative of TRAIL-induction in AsPC-1 cells treated with 0.5 µM or 5 µM ONC201 or ONC212 are shown. **E.** HPAF-II cells were treated with different doses of ONC201 or ONC212. **F.** The representative percentage of cells in sub-G1 phase is graphically represented. **G.** HPAF-II and AsPC-1 cells were treated at different durations of time with 5 µM ONC201 or ONC212. Indicated markers of apoptosis were subsequently assessed by western blot analysis. **H.** Effects on anti-apoptotic markers were measured 72 hours post ONC201 or ONC212 treatment (20 µM) in the indicated cell lines.

It was previously shown that following ONC201 treatment, cells first arrest in the G1-phase (by 24 hours) and then proceed to apoptosis (by 48-72 hours). However, a subset of cell lines do not undergo apoptosis, and instead the arrest in the G1-phase persists at 72 hours post-treatment [22, 25]. We evaluated the cell cycle status of pancreatic cancer cell lines following treatment with ONC201 or ONC212. Both compounds induced apoptosis only in two out of the seven cell lines tested as measured by increased sub-G1, 72 hours post treatment (Figure 2A). The other four cell lines had increased fraction of cells in the G2-M phase and one cell line (BxPC3) was arrested in the G1-phase (Figure 2B, 2C and Supplementary Figure 2A).

Induction of apoptosis in the sensitive pancreatic cancer cell lines was initiated by upregulation of TRAIL surface expression (Figure 2D). This upregulation of TRAIL further engaged the cell-extrinsic pathway of apoptosis as measured by increased cleaved caspase 8 (CC8) levels (Figure 2E). Consistent with our findings in cell proliferation assays, ten-times less ONC212 than ONC201 was needed for increasing cell-surface TRAIL expression (Figure 2D) and for inducing apoptosis (Figure 2E, 2F and Supplementary Figure 2B) in the sensitive cell lines. In addition, induction of apoptosis by ONC212 was an earlier event than ONC201. This is documented through observations of increased levels of CC8, CC3 and cleaved PARP at 24 hours post-ONC212 treatment compared to a later time point of 48 hours post-ONC201 treatment (Figure 2G). Lastly, treatment with ONC201 and ONC212 reduced the expression of anti-apoptotic markers such as XIAP and MCL-1, but their reduction was not sufficient to promote apoptosis in the drug-resistant cell lines (Figure 2H). In summary, most human pancreatic cancer cell lines arrest in S-G2/M phases and do not undergo apoptosis when treated with either ONC201 or ONC212. However, ONC212 induces apoptosis at an earlier time point and at a lower concentration than ONC201 when used for the treatment of the two drug-sensitive cell lines.

### ONC212 shows improved efficacy *versus* ONC201 against human pancreatic cancer xenograft models

**Figure 3:**
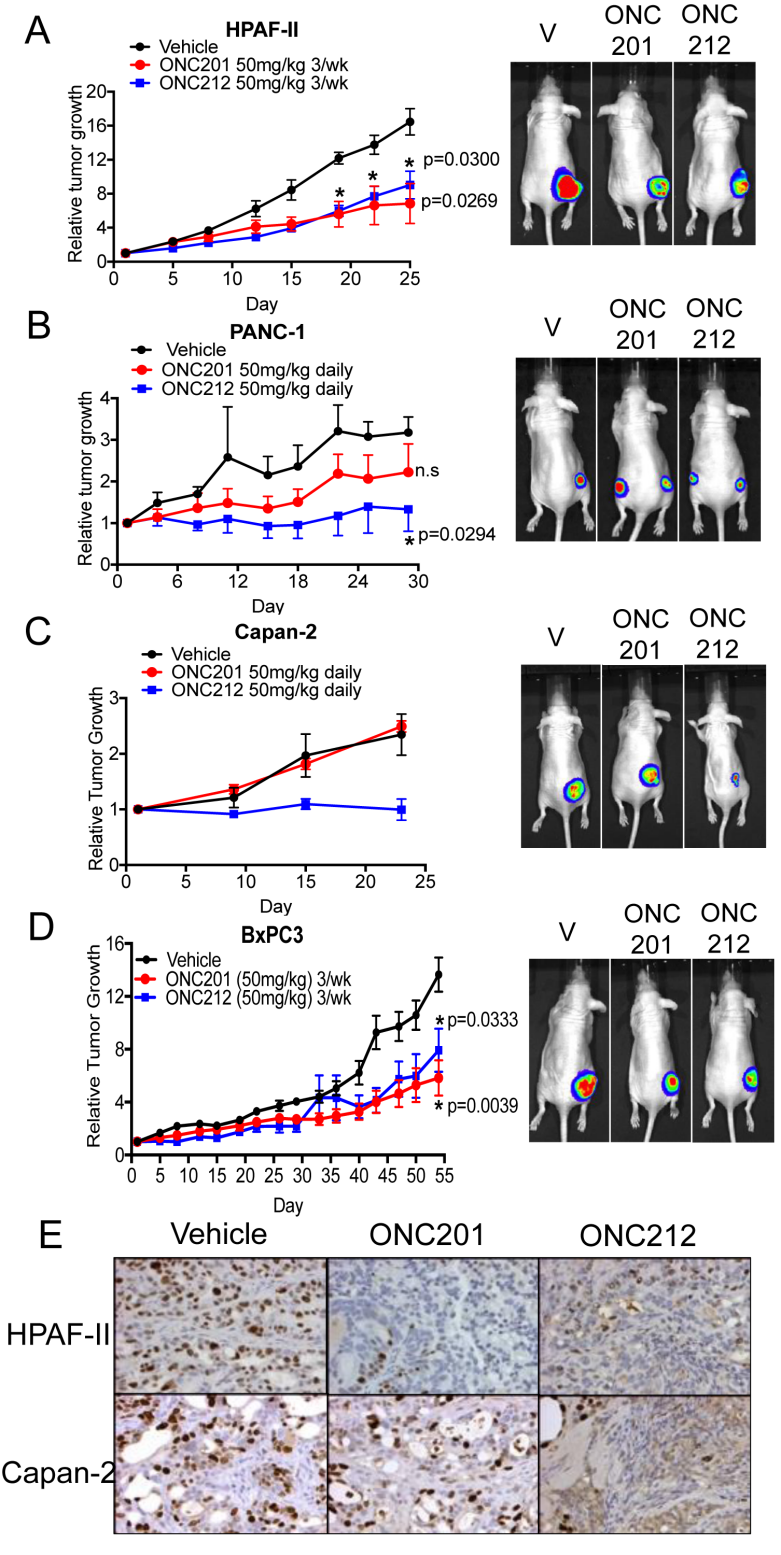
ONC212 shows improved efficacy *versus* ONC201 in human pancreatic cancer xenograft models Single-agent efficacy of ONC201 or ONC212 was investigated in four different *in vivo* models of pancreatic cancer. Doses of 50 mg/kg of either ONC201 or ONC212 were administered either daily (**B.** PANC-1, **C.** Capan-2) or three-times a week (**A.** HPAF-II, **D.** BxPC3) by oral gavage. (Left, **A.**-**D.**). Representative graphs of tumor growth over time. (Right, **A.**-**D.**) Bioluminescence imaging was performed at the end of each experiment. Representative bioluminescence images for each treatment cohort. **E.** Tumor sections from ONC201- or ONC212-treated mice were assessed for the proliferation marker Ki67. Representative Ki67 images of HPAF-II and Capan-2.

Following the observation that ONC212 exhibits more potent anti-proliferative or pro-apoptotic effects *versus* ONC201 *in-vitro*, we next tested the efficacy of ONC212 in comparison to ONC201 *in-vivo* using xenograft models of human pancreatic cancer. We compared the single agent efficacy of ONC201 and ONC212 using four different xenograft models (Figure 3). In two out of the four models tested, ONC212 treatment exhibited significantly greater growth inhibition in comparison to ONC201 (Figure 3B and 3C). More specifically, in the PANC-1 and Capan-2 xenograft models, daily dosing of 50 mg/kg ONC201 showed no significant growth inhibition, but there was a significant growth inhibitory effect using daily dosing of 50 mg/kg of ONC212 (Figure 3B and 3C). In HPAF-II and BxPC3 xenograft models, both ONC201 and ONC212 had comparable anti-tumor effects when mice were treated with either drug. A dose of 50 mg/kg of ONC201 or ONC212 administered three-times a week was sufficient to lead to significant growth inhibition of tumors compared to the control group for these two models (Figure 3A and 3D). Ki67 immunohistochemical staining demonstrated that both ONC201 and ONC212 treated tumors showed reduced proliferation in the HPAF-II model. However, only ONC212 reduced proliferation of Capan-2 tumors as compared to control-treated tumors (Figure 3E and Supplementary Figure 3). These results demonstrate the superior efficacy of ONC212 over ONC201 using *in-vivo* models of pancreatic cancer.

### ONC201 and ONC212 activate different branches of the unfolded protein response (UPR) in different cell lines leading to cell survival or apoptosis

**Figure 4:**
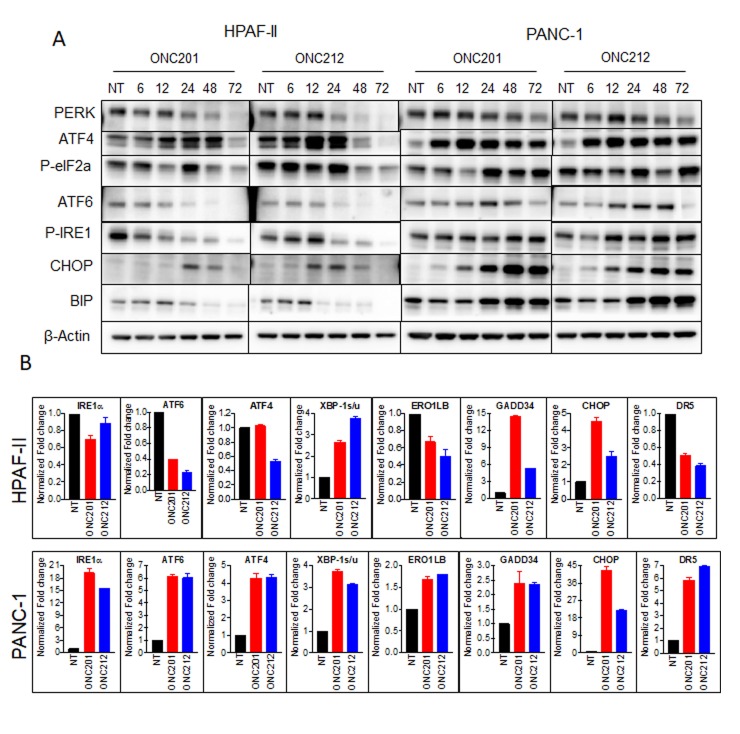
ONC201 and ONC212 activate different branches of the UPR in different cell lines leading to survival or apoptosis **A.** ONC201/ONC212-sensitive cell line HPAF-II and -resistant cell line PANC-1 were treated with the indicated doses of ONC201 or ONC212. Expression levels of Unfolded Protein Response (UPR) proteins were measured by western blot analysis 72 hours post-treatment. **B.** qRT-PCR was performed to measure changes in UPR genes 48 hours post ONC201 or ONC212 treatment (5 µM-HPAF-II; 20 µM-PANC-1).

It was previously shown that ONC201 induces the integrated stress response (ISR) [25, 29]. When cells undergo stress, unfolded proteins accumulate in the endoplasmic reticulum (ER). Subsequently, the unfolded protein response (UPR) is activated to enable the cells to either resolve the stress or initiate apoptosis. We hypothesized that the differences between cells that survive or proceed to apoptosis following treatment with ONC201 or ONC212 may be due to the activation of differential UPR branches, which subsequently influence cell fate. To test this hypothesis, we analyzed the expression and activation levels of a panel of UPR proteins by western blot analysis (Figure 4A), qRT-PCR (Figure 4B) and gene expression profiling (Supplementary Figure 4). Substantial differences between HPAF-II cells that undergo apoptosis, and PANC-1 cells that survive were observed following treatment with ONC201 or ONC212. More specifically, western blot analysis showed that in the HPAF-II cell line, ATF4 and phosphorylated EIF2α were upregulated as early as 6-12 hours post ONC201 or ONC212 treatment. Subsequently, CHOP was induced by 12 hours post ONC212 or by 24 hours post ONC201 treatment. PERK, phospho-IRE1α, and GRP78/BIP were downregulated. By contrast, PANC-1 cells upregulated ATF6, phospho-IER1α and BIP in addition to up-regulating ATF4 and activation of EIF2α 12-24 hours post ONC201 or ONC212 treatment. CHOP was substantially more up-regulated in PANC-1 cells compared to HPAF-II (Figure 4A). Although CHOP is known to trigger cell death following ER stress [30], PANC-1 cells survived probably due to the increased levels of the ER chaperone BIP, which was sufficient for survival. These results were confirmed by qRT-PCR analysis of samples 48 hours post treatment with ONC201 and ONC212 (Figure 4B). At the gene transcription level, significant up-regulation of GADD34, CHOP and activation of XBP-1 were observed in both PANC-1 and HPAF-II cells. No significant changes or down-regulation were observed in the transcript levels of ATF6, IRE1α and BIP genes in HPAF-II cells. However, all UPR genes, including target genes ERO1LB and DR5, were up-regulated in the PANC-1 cell line following treatment with either ONC201 or ONC212 (Figure 4B). Interestingly, although the pro-apoptotic proteins CHOP, GADD34 and DR5 were induced in PANC-1 cells, the cells survived. Gene expression analysis of the UPR pathway confirmed these results and suggested ER chaperones in addition to BIP that might be involved in the survival process including calnexin and calreticulin (Supplementary Figure 4).

### Surviving cells following treatment with ONC201 or ONC212 facilitate the UPR to up-regulate the ER chaperone BIP/GRP78

**Figure 5:**
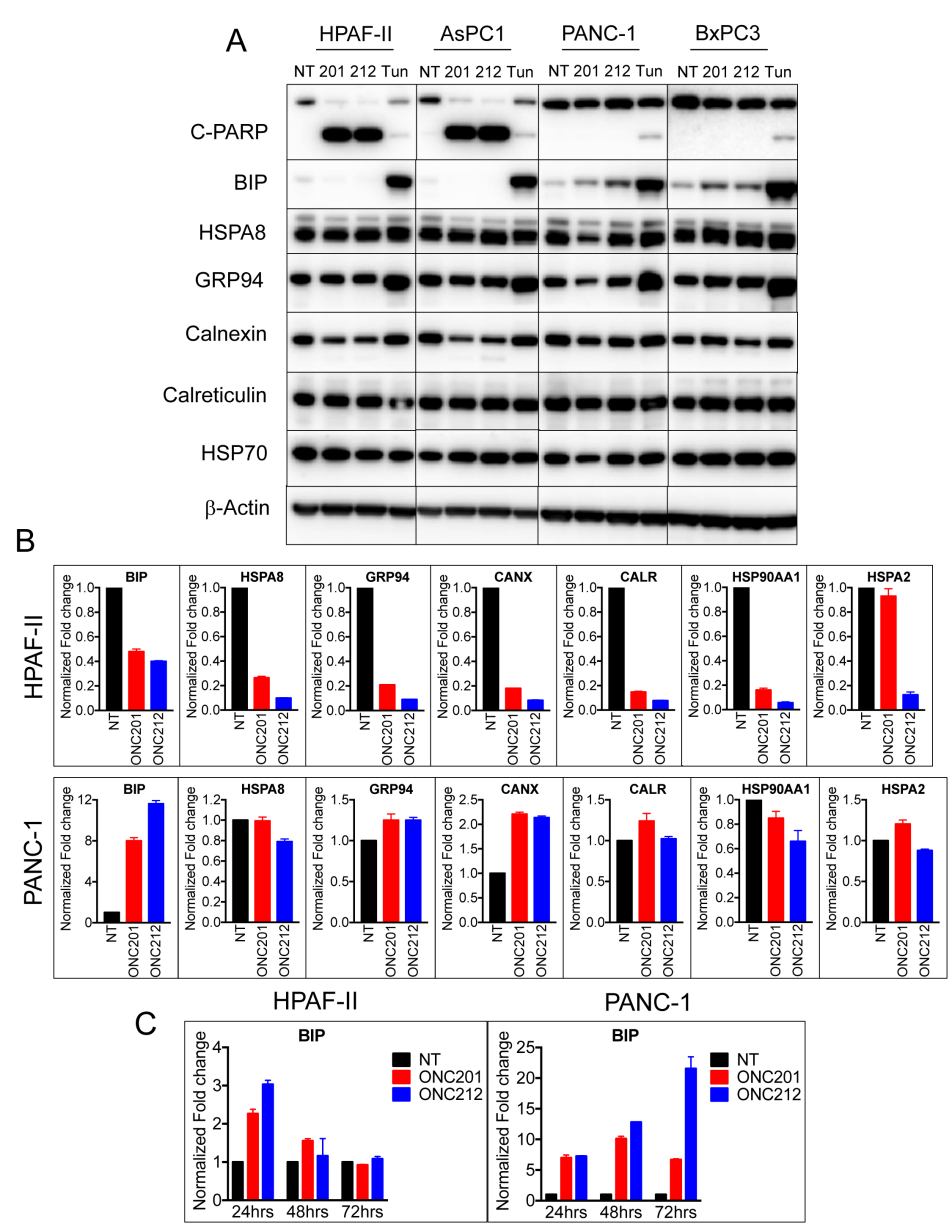
Surviving cells following treatment with ONC201 or ONC212 facilitate the UPR to upregulate the ER chaperone GRP78/BIP **A.** ONC201/ONC212-sensitive cell lines HPAF-II and AsPC-1 and -resistant cell lines PANC-1 and BxPC3 were treated with 20 µM of either ONC201 or ONC212 or tunicamyicin. Expression levels of the indicated chaperones were measured by western blot analysis 48 hours post-treatment. **B.** qRT-PCR analysis indicating changes in expression of ER chaperones is shown 48 hours post ONC201 or ONC212 treatment (5 µM-HPAF-II; 20 µM-PANC-1). **C.** qRT-PCR analysis of BIP expression over time is shown in HPAF-II and PANC-1 cell lines treated with 20 µM ONC201 or ONC212.

To test if ER chaperones were involved in the survival mechanism post ONC201 or ONC212 treatment, we monitored the expression of a panel of ER chaperones by western blot and qRT-PCR analysis (Figure 5). Sensitive cells (HPAF-II, AsPC-1) and resistant cells (PANC-1, BxPC-3) were treated with ONC201 or ONC212 for 48 hours and the ER stress inducer tunicamycin was used as a control. Western blot analysis demonstrated a correlation between increased levels of BIP and survival of cells to treatment with ONC201 and ONC212. PANC-1 and BxPC3 cells that survive treatment with ONC201 and ONC212, showed increased levels of BIP. HPAF-II and AsPC-1, cells that undergo apoptosis, did not express any detectable levels of BIP following treatment with ONC201 and ONC212 but demonstrated increased levels of BIP following treatment with tunicamycin. Other chaperones tested including HSPA8, GRP94, calnexin, calreticulin and HSP70 were either un-changed or down-regulated following treatment with ONC201 or ONC212 in all cell lines tested (Figure 5A). Similar results were obtained by qRT-PCR analysis 48 hours post treatment with ONC201 or ONC212. Treated HPAF-II cells showed decreased transcript levels of all chaperones, while in PANC-1 cells, BIP and calnexin were induced 8-12 fold and 2-fold respectively (Figure 5B). Time course analysis of BIP transcript showed transient 2-3 fold increases in BIP mRNA at 24 hours post treatment with ONC201 or ONC212 in HPAF-II cells. The induction of BIP in PANC-1 cells started at about a 6-fold increase at 24 hours and persisted at 48 and 72 hours post treatment (Figure 5C). This observation suggests that the ER chaperone GRP78/BIP is involved in the survival mechanism of PANC-1. In order to further demonstrate the importance of BIP in PANC-1 survival after treatment with ONC201 and ONC212, we treated the cells with or without gene silencing of BIP using siRNA. Although BIP was 80-90% knocked down, PANC-1 cell did not become sensitive to treatment with ONC201 or ONC212 and did not undergo apoptosis (data not shown). This could be due to compensation by other ER chaperones as indicated in the microarray analysis (Supplementary Figure 4).

### ONC201 and ONC212 show synergistic potential with IGF1-R inhibitor AG1024 in pancreatic cancer cell lines and *in vivo*

**Figure 6:**
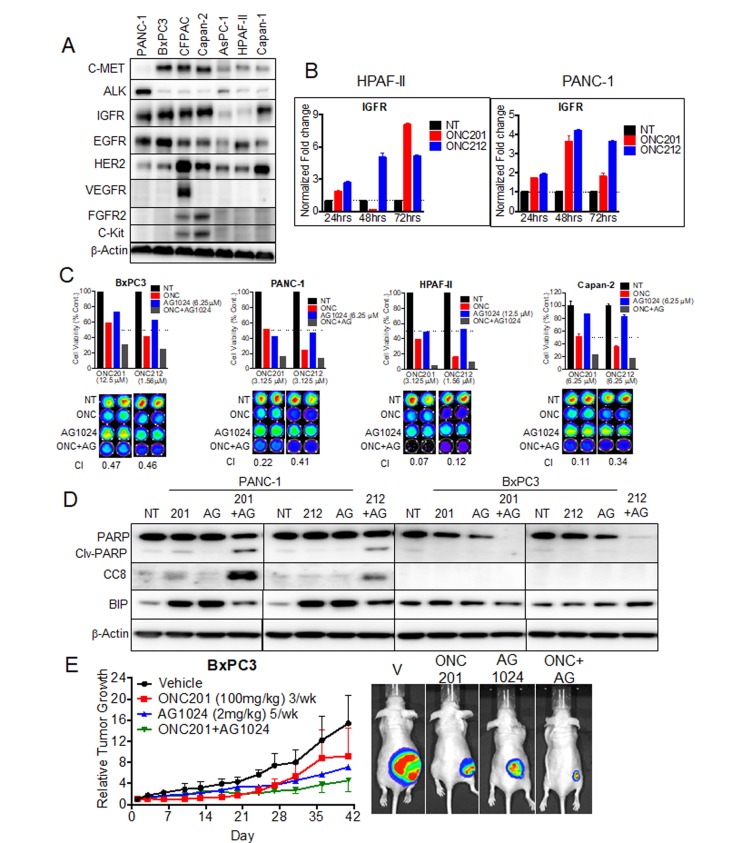
ONC201 and ONC212 show synergistic anti-cancer activity with IGF1-R inhibitor AG1024 in pancreatic cancer cell lines **A.** Representative western blot for expression of RTKs assessed from whole cell lysates of seven pancreatic cancer cell lines. **B.** qRT-PCR analysis of IGF1-R expression over time for the HPAF-II and PANC-1 cell lines, treated with 20 µM ONC201 or ONC212. **C.** Combination experiments with IGF1-R inhibitor AG1024 (0-25 µM) were assayed in both ONC201/ONC212-sensitive cell line HPAF-II and -resistant cell lines (PANC-1, BxPC3 and Capan 2). ONC201 and ONC212 were used in the ranges of 0-100 µM and 0-50 µM respectively. Synergy for AG1024-ONC201 or -ONC212 combinations was measured by cell viability 72 hours post-treatment using the CellTiter-Glo assay. (Top) Indicated graphs represent a selected synergistic dose of AG1024 and ONC201 or ONC212. (Bottom) CellTiter-Glo image and CI index (Bottom) for all four cell lines tested. **D.** PANC-1 and BxPC3 were treated with either 20 µM ONC201 or ONC212 or 5 µM AG1024 or combinations as indicated. Representative western blot for markers of apoptosis is shown 72 hours post-treatment. **E.** Combination efficacy of ONC201 (100 mg/kg, 3/week) and AG1024 (2 mg/kg, 5/week) was investigated in the BxPC3 xenograft model. (Left) Representative graphs of tumor growth over time are shown. (Right) Bioluminescence imaging was performed at the end of the experiment. Representative bioluminescence images for each treatment cohort are shown.

Receptor tyrosine kinases (RTKs) are overexpressed in a significant number of cancers including pancreatic cancer. The abnormal expression of these receptors has been associated with the development and progression of the cancer. We previously tested if ONC201 inhibits the activation of a panel of RTKs including EGFR, c-MET, VEGF and IGF1-R that were induced by the corresponding growth factors (data not shown). ONC201 did not directly inhibit the activation of any of the RTKs tested. To test for a correlation between sensitivity to ONC201 or ONC212 and expression of RTKs, we screened for protein expression of a panel of RTKs in all seven pancreatic cancer cell lines used in this study (Figure 6A). In this analysis, expression level of IGF1-R showed the highest correlation to sensitivity to either ONC201 or ONC212. The two cell lines that undergo apoptosis (HPAF-II and AsPC-1) expressed low levels of IGF1-R while the other cell lines that arrest and survive, expressed high levels of IGF1-R (Figure 6A). Analysis of IGF1-R transcription following treatment with ONC201 and ONC212 demonstrated an increasing expression with time in both IGF1-R low expressing cells (HPAF-II) and high IGF1-R expressing cells (PANC-1) (Figure 6B).

We hypothesized that inhibiting IGF1-R might sensitize cells to ONC201 or ONC212 and show a synergistic effect. To test this hypothesis, we first performed an *in-vitro* cell viability assay using combinations of ONC201 or ONC212 with the IGF1-R inhibitor AG1024 (Figure 6C). AG1024 was synergistic with either ONC201 or ONC212 in the four cell lines tested (Figure 6C). Combination indices (CIs) of < 1 (indicating synergy) were observed at the ONC201/212-AG1024 combination doses tested for each cell line. In addition, PANC-1 cells underwent apoptosis only when treated with combination of ONC201 or ONC212 with AG1024 as demonstrated by cleaved PARP and CC8 expression in western blot analysis (Figure 6D). BxPC3 cells did not show any marker of apoptosis following combination treatment but showed decreased levels of total PARP. This observation suggests that BxPC3 treated with the combination of drugs might go through a different cell death pathway (Figure 6D). Lastly, an initial *in-vivo* experiment showed potential synergy between ONC201 and AG1024 (Figure 6E).

### ONC212 shows synergistic potential with chemotherapeutic drugs for pancreatic cancer and crizotinib

**Figure 7:**
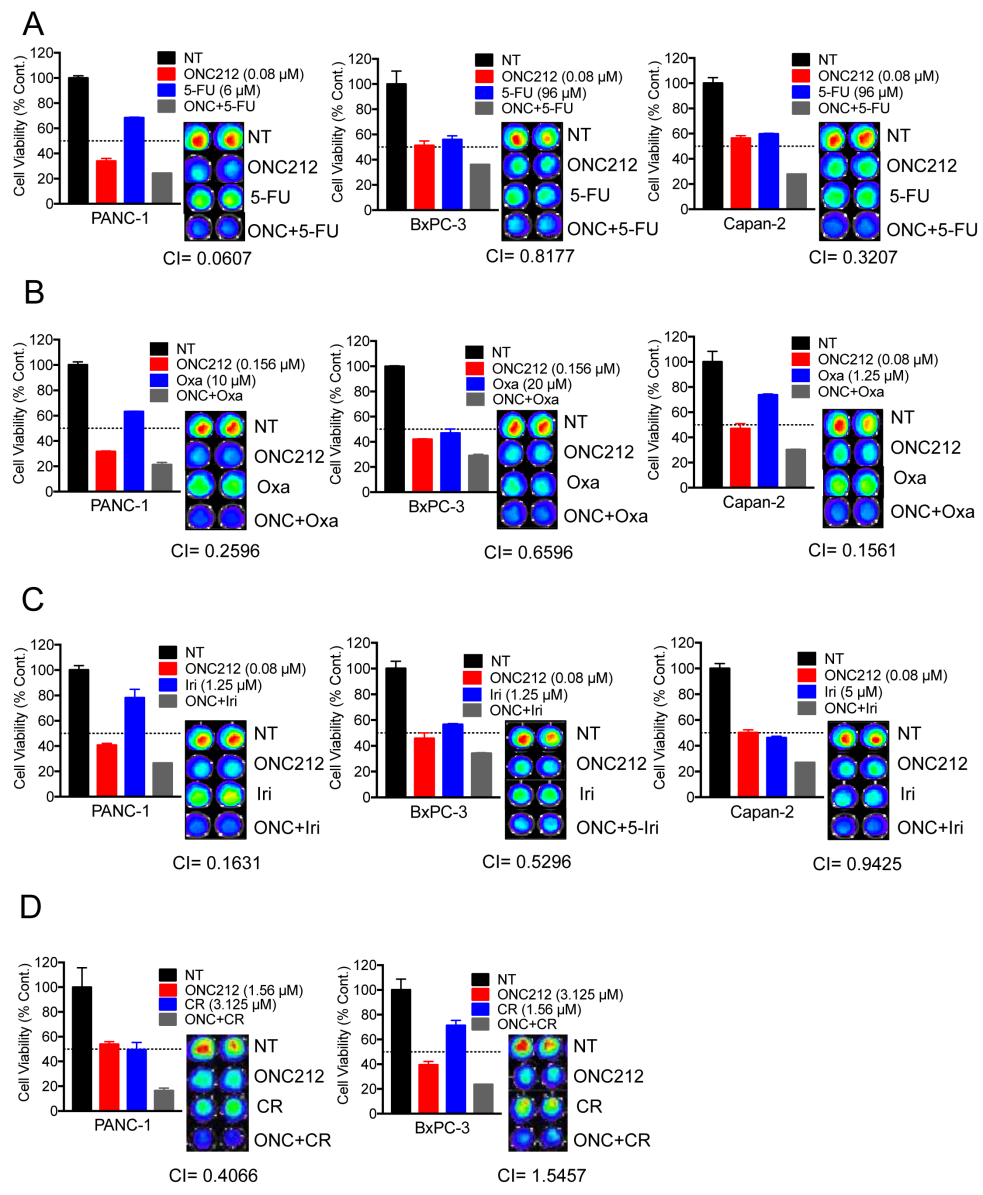
ONC212 shows synergistic anti-cancer activity with 5-fluorouracil, oxaliplatin, irinotecan or crizotinib against human pancreatic cancer cell lines Combination experiments with 5-fluorouracil (0-384 µM), oxaliplatin (0-20 µM) and irinotecan (0-10 µM) were assayed using resistant cell lines (PANC-1, BxPC3 and Capan 2), with ONC212 given in ranges of 0-5 µM. Chemotherapeutic drug synergy with ONC212 was measured by cell viability 72 hours post-treatment using the CellTiter-Glo assay. (Left, **A.**-**C.**) Graphs represent a selected synergistic dose of ONC212 with the indicated drugs. (Right, **A.**-**C.**) Corresponding CellTiter-Glo image and CI index for all four cell lines tested. **D.** PANC-1 and BxPC3 were also treated with combinations of ONC212 (0-100 µM) and crizotinib (0-25 µM). Representative graphs and CI indices of selected combinations and doses of ONC212 and CR are shown.

Since ONC212 was more efficacious than ONC201 in a variety of *in-vitro* and *in-vivo* models, we aimed at evaluating its therapeutic potential in combination with front-line therapeutic drugs for pancreatic cancer. We performed cell viability assays using three cell lines in which ONC212 had an anti-proliferative effect but did not promote apoptosis (PANC-1, BxPC3 and Capan-2). Calculation of combination indices indicated that ONC212 is synergistic with 5-fluorouracil (Figure 7A), oxaliplatin (Figure 7B) and irinotecan (Figure 7C) in the three cell lines tested. Combination treatment with Gemcitabine did not show synergy (data not shown). In addition, since BxPC3 cells and PANC-1 cells highly expresses c-MET and ALK respectively (Figure 6A), we performed similar cell viability assays combining ONC212 with crizotinib. ONC212 was synergistic with crizotinib in PANC-1 cells but not in BxPC3 (Figure 7D). These cells also highly express EGFR and/or HER2 (Figure 6A). However, erlotinib and lapatinib showed only a mild effect in this assay and need to be further tested for their synergistic potential (data not shown).

In summary, ONC212 is a promising anti-cancer drug showing single agent efficacy both *in vitro* and *in vivo* models of human pancreatic cancer. Furthermore, combination of ONC212 with current front-line chemotherapy or selected targeted therapies (IGF1-R) shows potential for clinical translation.

## DISCUSSION

We evaluated the efficacy of imipridones ONC201 or ONC212 as single drugs or in combination with other drugs using cell lines and *in vivo* models of pancreatic cancer. In previous studies, ONC201 showed efficacy in multiple tumor types tested including initial results in patients [23]. Due to the lack of effective drugs to target pancreatic cancer and the imperative need to find new drugs, there is significant interest in evaluating the efficacy of ONC201 and the analogue ONC212 in pancreatic cancer. Indeed, both ONC201 and ONC212 showed an anti-proliferative effect in a large panel of pancreatic cancer cell lines with ONC212 having at least a ten-fold increased potency than ONC201 (Figure 1). It was previously shown that ONC201 induces surface TRAIL and promotes apoptosis through the extrinsic cell death pathway. However, in most pancreatic cancer cell lines tested, ONC201 did not induce surface TRAIL expression and the cells did not undergo apoptosis. Indeed, cells were arrested in G1 or G2-M in response to treatment. The minority of pancreatic cancer cell lines that underwent apoptosis followed the previously described mechanism of cell death with at least a ten-fold increased potency of ONC212 over ONC201 (Figure 2). Since ONC212 was superior to ONC201 using a variety of assays in a panel of *in-vitro* models, we evaluated the efficacy of ONC212 in comparison to ONC201 *in-vivo* using xenograft models of pancreatic cancer. In two out of four *in-vivo* models used, ONC212 was more efficacious than ONC201 (Figure 3), suggesting further exploration of the therapeutic potential of ONC212 for pancreatic cancer patients is warranted.

Resistance of pancreatic cancer to chemotherapy, immunotherapy, and other anti-cancer drugs is a major challenge [31]. The mechanism of resistance may be cell intrinsic or mediated by the tumor microenvironment. We were interested in understanding the resistance mechanism by which pancreatic cancer cells survive treatment with ONC201 or ONC212. To first look at the cell intrinsic mechanism of resistance we compared the cellular stress response between sensitive cells (cells that undergo apoptosis) to resistant cells (cells that survive but are cell cycle arrested). It was previously shown that both ONC201 and ONC212 induce cell stress. Since cell stress promotes the accumulation of unfolded proteins in the ER and triggers the UPR pathway, we investigated the three branches of the UPR following treatment with ONC201 or ONC212. The three branches of the UPR are mediated through three ER transmembrane receptors: PERK, ATF6 and IRE1α. Under normal conditions, these receptors are maintained in an inactive state through their association with the ER chaperone GRP78/BIP. Upon accumulation of unfolded proteins in the ER, BIP is dissociated from the receptors leading to their activation. The activation of the three UPR branches impedes protein synthesis, enhances the expression of ER chaperones and ER quality control proteins and activates the ER associated degradation (ERAD) pathway, all of which are important for restoring of the ER proteostasis. However, if ER proteostasis cannot be restored, then apoptosis is initiated. In Figure 4 we investigated the three branches of the UPR pathway in the sensitive cell line HPAF-II in comparison to the resistant cell line PANC-1. In response to treatment with ONC201 or ONC212, HPAF-II cells upregulated protein expression of ATF4, GADD34 and CHOP that are pro-apoptotic but the expression levels of the pro-survival chaperone BIP was reduced. Further, HPAF-II did not show substantial induction of the IREα and ATF6 branches of the UPR, which in part could explain lack of rescue from apoptosis. By contrast, in response to treatment with ONC201 or ONC212, PANC-1 cells up-regulated all three branches of the UPR including increasing expression of BIP that was sufficient to evade apoptosis (Figure 4). Since the UPR pathway is known to induce the expression of a panel of ER chaperones including BIP, GRP94, HSPA8, calnexin and calreticulin, we tested the expression levels of these chaperones in response to treatment with ONC201 or ONC212 in two drug-sensitive cell lines (HPAF-II and AsPC1) and two drug-resistant cell lines (PANC-1 and BxPC3). This analysis showed a correlation between resistance and increased expression of BIP. qRT-PCR and microarray analysis suggested a possible involvement of other chaperones including calnexin, calreticulin and HSPA8, but only BIP was elevated at the protein level as shown by western blot analysis (Figure 5 and Supplementary Figure 4). GRP78/BIP (also known as HSPA5) is a member of the HSP70 superfamily and is critical for the protein folding and maturation in the ER compartment. While normally residing in the ER, GRP78 is also found in other cellular compartments and in particular is elevated on the cell surface of cancer cells in response to cellular stress. As a pro-survival chaperone, increased levels of GRP78 were found to be involved in cell survival and proliferation in cancer [32, 33]. Elevated GRP78 expression increases chemoresistance and is associated with poor prognosis in patients with pancreatic cancer [34, 35]. Although there is no FDA-approved drug, targeting GRP78 in the clinic, there is evidence of therapeutic benefit in targeting GRP78 in combination with other treatments [36, 37]. Our results implicate the involvement of GRP78/BIP in drug-resistance to ONC201 or ONC212. Consequently, targeting GRP78 in addition to treatment with ONC212 might have therapeutic benefit for pancreatic cancer patients.

Receptor tyrosine kinases (RTKs) are essential for the growth, development and maintenance of many tissues in the human body. In cancer, RTKs are often over-expressed or constitutively active, thus promoting cell survival, proliferation and invasion [38]. Therefore, RTKs have been a major target for the development of small molecule drugs for cancer [39]. We investigated the protein expression levels of RTKs in the seven pancreatic cancer cell lines used in this study. Among RTKs tested, there was a correlation between high expression of IGF1-R and resistance to ONC201 or ONC212 (Figure 6A). Furthermore, treatment with ONC201 or ONC212 induced the transcription of IGF1-R (Figure 6B). There is evidence of abnormal, pro-tumorigenic and pro-survival IGF1-R signaling in several cancers including pancreatic cancer [40, 41]. Numerous studies show the involvement of IGF1-R in tumorigenesis and development of cancer drug resistance [40, 42, 43]. Targeting IGF1-R has previously shown therapeutic benefit by blunting pancreatic cancer growth and metastasis [9]. In addition, a recent study by Novosyadlyy *et al.* showed that IGF1 protects cells from ER stress-induced apoptosis, by increasing expression of GRP78/BIP and thereby increasing the adaptive capacity of the ER [44]. Another study by Pfaffenbach *et al.* pointed out GRP78/BIP as a novel downstream target of IGF1-R mediated signaling [45]. We hypothesized that targeting IGF1-R in combination with treatment with ONC201 or ONC212 could promote apoptosis as well as downregulate the expression of GRP78/BIP. Indeed, treatment with ONC201 or ONC212 in combination with the IGF1-R specific inhibitor AG1024 resulted in a synergistic anti-cancer effect and initiation of apoptosis in PANC-1 cells that survived each drug alone (Figure 6C and 6D). Combination of ONC201 or ONC212 plus AG1024, also attenuated the expression of GRP78/BIP as predicted. Our preliminary *in-vivo* study of the combination treatment of ONC201 and AG1024 further supports the rationale for combining IGF1-R targeted therapy with ONC201 or ONC212 (Figure 6E). Due to toxicity, IGF1-R inhibitors have limited use in the clinic. However, enormous efforts are underway to develop such therapies [46]. The synergy between IGF1-R inhibitor and ONC212 and the attenuation of BIP expression makes this combination a promising treatment for pancreatic cancer patients.

Since ONC212 showed notable pre-clinical efficacy against pancreatic cancer, both as a single agent as well as in combination with an IGF1-R inhibitor, we wanted to test its potential in combination with FDA-approved drugs for pancreatic cancer. *In-vitro* testing by cell viability assays revealed synergy between ONC212 and 5-fluorouracil, oxaliplatin and irinotecan (Figure 7). In addition, combining ONC212 with small molecules could be beneficial depending on the mutation or abnormal expression of specific targets in individuals. For example, ALK overexpressing cell line, PANC-1, showed a striking synergy when ONC212 was added with crizotinib (Figure 7D), which is currently approved for ALK expressing locally advanced or metastatic non-small cell lung cancer [47]. Identifying pancreatic cancer patients with similar mutations or additional RTK mutations may help tailor ONC212 combination treatments for such individuals. The benefit of combination therapy is well established in many tumor models as well as in the clinic and should be explored for ONC212 for the treatment of pancreatic cancer.

In summary, we have demonstrated the *in-vitro* and *in-vivo* efficacy of ONC212, a novel member of the imipridone class of anti-cancer agents. Our work also provides pre-clinical data for combining ONC212 with approved chemo and targeted therapies, with imminent translational potential. Our ongoing work is aimed at expanding the tumor types that could benefit from ONC212 treatment and testing the proposed combinations in different *in vivo* models of pancreatic cancer.

## MATERIALS AND METHODS

### Cell culture and reagents

All pancreatic cancer cell lines were obtained from American Type Culture Collection (ATCC). The cell lines were maintained in DMEM supplemented with 10% FBS and 1% penicillin-streptomycin solution. All low passage patient derived pancreatic cancer cell lines were maintained in RPMI supplemented with 10% FBS and 1% penicillin-streptomycin, glutamine and insulin solutions. Cell lines were mycoplasma free and were authenticated routinely. ONC201 and ONC212 were obtained from Oncoceutics, Inc. AG1024 was purchased from Selleckchem (Cat no. S1234). The chemotherapeutic agents oxaliplatin, 5-fluorouracil and irinotecan were obtained from the Fox Chase Cancer Center infusion pharmacy.

### Cell proliferation assays

Cell proliferation was measured by both CellTiter-Glo^®^ and MTT assays. Briefly, 10,000 cells were seeded in a 96 well plate overnight. Cells were treated with either single agent or combination at indicated concentrations of ONC201, ONC212 and other drugs. At 72 hours post-treatment luminescent-based cell viability was determined using Cell-Titer Glo (CTG) assay according to the manufacturer’s instructions (Promega). For MTT assay, cells were incubated with 20 µL of 5 mg/mL MTT substrate for 4 hours and later dissolved using MTT solvent (4 mM HCl, 0.1% Nondet P-40 (NP40) in isopropanol). Absorbance was measured at 575 nM. Percent of cell viability/proliferation was determined by normalizing luminescence/absorbance signal to untreated control well. All treatments were done in triplicates and reported as % Viability + SEM and % Proliferation + SEM. Dose response curves were generated to calculate the half-maximal growth inhibition concentration (GI_50_) in GraphPad Prism version 6.07. Combination indices were calculated using Compusyn software (ComboSyn, Inc.).

### Genomics of drug sensitivity in cancer (GDSC) cell line screening

A panel of 33 pancreatic cancer cell lines was used to test the effect of ONC201 and ONC212 (78 nM-20 µM) by cell viability assay, 72 hours post-treatment. Dose response curves were used to calculate IC50 and Area Under Curve (AUC) [48, 49].

### Colony formation assay

Colony formation assays were performed by seeding 0.2 X 10^6^ cells/well in a 6-well plate and treatment with indicated doses of ONC201 or ONC212. At 72 hours post-treatment, cells were harvested and 500 cells per treatment group were plated in drug-free media in triplicate for colony formation. Colonies were stained with 0.25% crystal violet on Day 10, imaged, counted and reported as number of colonies +SEM.

### Cell cycle analysis

All pancreatic cancer cell lines were treated with ONC201 or ONC212 at the indicated doses and time-points. Post-treatment, both floating and adherent cells were collected, fixed in 70% ethanol and stained with propidium iodide in the presence of ribonuclease A. Flow cytometric data was collected using an EPICS Elite flow cytometer (Beckman-Coulter). The sub-G1 fraction (apoptotic) was quantified, and analysis was performed to quantify the distribution of cells in G1, S and G2-M phases of the cell cycle utilizing FlowJo software (FlowJo, LLC).

### Cell surface staining for TRAIL

Cells were harvested using enzyme-free cell dissociation buffer (Life Technologies). Cells were washed with FACS buffer (PBS with 1% FBS and 0.1% sodium azide) and stained with conjugated antibodies against TRAIL (Biolegend, 308205). Flow cytometry data was collected using LSR II flow cytometer (BD Biosciences). Flow-Jo software was used to exclude doublets and analyze data.

### Immunoblotting

Cells were washed with PBS and lysed with RIPA buffer (Sigma) supplemented with protease inhibitor (Roche) and phosphatase inhibitor (Roche). The protein concentration of cell lysates was determined using BCA Protein Assay Kit (Thermo Fisher Scientific) and equal amounts of proteins were loaded onto NuPAGE 4-12% Bis-Tris gel (Invitrogen). The separated proteins were transferred to PVDF membrane. After blocking with 5% bovine serum albumin in PBS, the membranes were incubated with primary antibody diluted 1:1000 in PBS/BSA overnight at 4°C. Membranes were then incubated with the appropriate secondary antibodies labeled with horseradish peroxidase followed by chemiluminescence detection using ECL Reagents (Thermo Fisher Scientific). The primary antibodies used in this study were as follows: antibodies against c-PARP (Cell Signaling Technology (CST), cat. no. 9546), CC3 (CST Asp175, cat. no. 9661), CC8 (CST Asp391, cat. no. 9496), ATF-4 (CST cat. no., 11815), ATF-6 (CST cat. no., 65880S), CHOP (CST cat. no., 2895S), BIP (CST cat. no., 3177S), GRP94 (CST cat. no., 2104), XBP-1s (CST cat. no., 12782), XIAP (CST cat. no., 2042S), MCL-1 (CST cat. no., 94296S), Cyclin D1 (CST cat. no. 92G2), and phospho-Rb (S780) (CST cat. no., 9307), BCL_xL_ (CST cat. no., 2764S), PERK (Santa Cruz cat. no., sc-377400), p-EIF2α (abcam cat. no., ab32157), p-IRE-1α (abcam cat. no., ab124945), HSPA8 (CST cat. no., 8444S), Calnexin (CST cat. no., 2679S), Calreticulun (CST cat. no. 12238S), HSP70 (CST cat. no., 4872S), c-MET (cat. no., 4560), ALK (abcam cat. no., ab140534), IGFR (CST cat. no., 3027S), EGFR (Santa Cruz cat. no., sc-03), HER2 (CST cat. no., 2242S), VEGFR (CST cat. no., 2479S), FGFR2 (CST cat. no., 11835S), c-Kit (CST cat. no., 3074S) Actin (Sigma, cat. No., A5441). Secondary antibodies acquired from Jackson Laboratories were horseradish-peroxidase conjugated.

### Immunohistochemistry

For immunohistochemical analyses, excised tumors were fixed in formalin overnight. Paraffin embedding and serial sectioning of slides were performed by the Fox Chase Cancer Center Histology Core Facility. Slides were de-waxed in xylene and hydrated in a decreasing gradient of ethanol. Antigen retrieval was carried out by boiling in 10 mM citric acid (pH 6.0) for 6 min. Samples were blocked with horse serum (Vector Laboratories) and incubated with primary antibody against Ki67 overnight at 4°C in a humidified chamber. Incubation with biotinylated secondary antibody and DAB deposition were carried out according to the manufacturer’s protocol (Vector Laboratories). Samples were counterstained with hematoxylin, rinsed in distilled water, de- hydrated, cleared with xylene, and mounted with Cytoseal XYL. Images were recorded on an Axioskop microscope (Zeiss) with QCapture software (QImaging).

### Quantitative RT-PCR (qRT-PCR)

Total RNA was isolated using the Quick-RNA™ MiniPrep kit (Zymo Research, Irvine, CA). 1 µg of total RNA from each sample was subjected to cDNA synthesis using SuperScript^®^ III Reverse Transcriptase kit (Life technologies, Grand Island, NY), for detection of indicated genes and housekeeping gene- GAPDH. Each cDNA sample was amplified using Power SYBR Green (Applied Biosystems, CA). The relative expression of the reported genes was determined using real-time PCR performed on Applied Biosystems 7900HT Fast Real-Time PCR system. The primers used are listed in Supplementary Table 1. Fold-change for every gene was calculated by 2^-ΔΔ^Ct method.

### Microarray analysis

A total of 0.3 X10^6^ HPAF-II, PANC-1 and BxPC3 cells were plated and treated with 20 μM ONC201 or ONC212 for 48 hours. Cells were harvested and RNA was isolated using Quick-RNA™ MiniPrep kit (Zymo Research, Irvine, CA) according to manufacturer’s instructions and submitted to the FCCC Genomics Facility for gene expression analyses by microarray. Quality of RNA specimens was determined by Agilent Bioanalyzer RNA kits and RNA was amplified and labeled using the low RNA input linear amplification kit (Agilent, Santa Clara, CA, USA). Labeled cDNA targets were hybridized onto Affymetrix Human Gene 2.0-ST array. Raw data were quantile-normalized using RMA method [50]. Ratios of gene expression level in each individual drug treated *versus* control were calculated and those with at least 50% up or down were considered genes of interest.

### Establishment of stably expressing luciferase cells

Cell lines stably expressing luciferase were established to monitor tumor growth by whole body bioluminescence imaging. Stable cells were generated with lentiviral particles using the luciferase containing pRL-SV40-Luc vector. Lentiviral particles were generated by transfecting pRL-SV40-Luc with packaging plasmids pCMV-VSV-G and pCMV delta R8.2 in HEK293T cells in ratio 2:1:1. At 72 hours post-transfection, lentiviral particles were collected and applied to cells in 1:1 ratio with antibiotic-free media. Stable clones were selected with puromycin: PANC-1 (6 µg/mL), HPAF-II (3 µg/mL), BxPC3 (2 µg/mL) and Capan-2 (3 µg/mL).

### *In-vivo* studies

The experimental mouse protocol was approved by the Institutional Animal Care and Use Committee (IACUC) of Fox Chase Cancer Center. Mouse experiments were free of pathogens including *mouse hepatitis virus* and *c. bovis*. Six- to seven-week-old female athymic nu/nu mice were purchased from Taconic (Hudson, NY) and were acclimated for 1 week on arrival to the animal facility before *in-vivo* study was initiated. A total of 3-5 × 10^6^ luciferase-expressing cells were suspended in 50 μl of PBS mixed with 50 μl of Matrigel (BD Biosciences) and subcutaneously injected into the rear flanks of the mice. When tumor volume reached an average of 100-150 cm^3^, mice were randomly assigned to the indicated control or treatment groups. ONC201 and ONC212 were delivered in a solution of 10% DMSO, 20% Kolliphor^®^ EL (Sigma cat. no. C5135) and 70% PBS by oral gavage. AG1024 was delivered in PBS containing less then 0.1% DMSO by daily intra-peritoneal injection. The length (L) and width (W) of the tumors were measured 1-2 times a week using a digital caliper, and the volume of the tumor was calculated using the formula: 0.5*L*W^2. Mice were also weighed once a week to monitor signs of drug toxicity. For whole body bioluminescence imaging, mice were injected with 100 microliters of D-Luciferin (3.75 µg/µL) (Gold BioTechnology) and were anesthetized with isoflurane attached to a nose-cone mask within Xenogen IVIS 200 imaging system. At the end of the experiment, mice were sacrificed. Tumor and organs were harvested for further IHC, western blot and RNA analysis of relevant markers.

### Statistics

All statistical analyses were conducted using GraphPad Prism 6. Data are presented as means ± standard error of mean from at least 3 replicates. The Student’s two-tailed T test in GraphPad Prism 6 was used for pairwise analysis. Statistically significant changes are indicated in the figures with p-values.

## SUPPLEMENTARY MATERIALS FIGURES AND TABLES


