# Supplementary material for: Anti-pancreatic cancer activity of ONC212 involves the unfolded protein response (UPR) and is reduced by IGF1-R and GRP78/BIP
Source: Oncotarget. 2017 Sep 12;8(47):81776–93. doi: 10.18632/oncotarget.20819 (PMC5669847; doi:10.18632/oncotarget.20819)
Supplement: Supplementary file 1 [file oncotarget-08-81776-s001.pdf]

Anti-pancreatic cancer activity of ONC212 involves the unfolded protein response (UPR) and is reduced by IGF1-R and GRP78/BIP

SUPPLEMENTARY MATERIALS

Supplementary Table 1: GI_{50} doses of ONC201 and ONC212 in a panel of seven human pancreatic cancer cell lines and nine low-passage PDX pancreatic cancer cell lines

Cell Line	GI_{50} (μM)		PDX Cell line	GI_{50} (μM)	
	ONC201	ONC212		ONC201	ONC212
BxPC3	12.5	0.25	PNX0001	70	14
PANC-1	3.125	0.17	PNX0050	10	2
Capan-2	6.25	0.18	PNX0027	1.5	0.28
CFPAC-1	7	0.19	PNX0015	3.75	0.4
Capan-1	3	0.2	PNX0017	50	0.55
HPAF-II	4.5	0.15	PNX0007	100	16
AsPC-1	4	0.14	PNX0016	3	20
			PNX0029	100	25
			PDSE-JH101	18	9

A

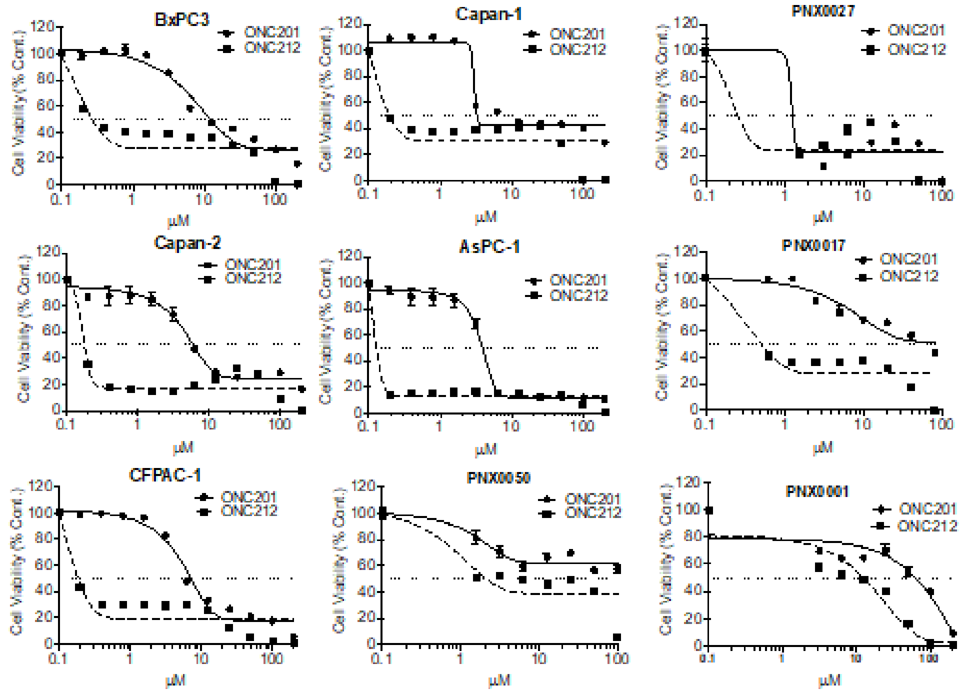

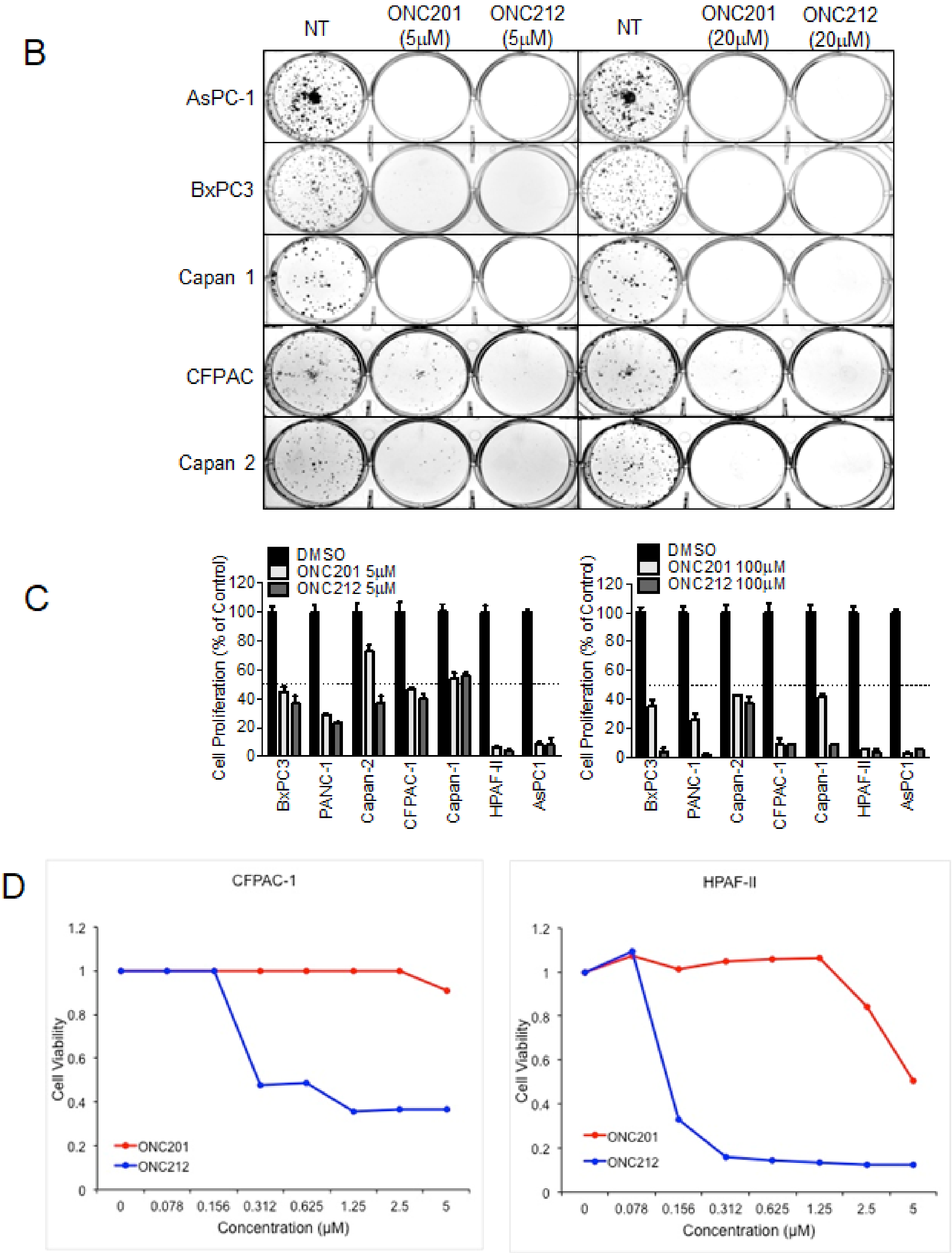

Supplementary Figure 1: (A) Dose-response curves for five pancreatic cancer lines (BxPC3, Capan-2, CFPAC-1, Capan- 1 and AsPC-1) and PDX cell lines treated with 0-100 µM of ONC201 or ONC212**.** (**B**) Representative image of colony formation in ONC201/ONC212 treated pancreatic cancer cell lines. (**C**) Short-term cell proliferation measured by MTT assay 72 hours post ONC201 or ONC212. Graphs represent percent proliferation normalized to untreated control at 5 µM (Left) and 20 µM (Right) doses of ONC201 or ONC212. (**D**) Cell viability assays comparing ONC201 and ONC212 in selected pancreatic cancer cell lines. The data in Panel D was generated through the GDSC screen.

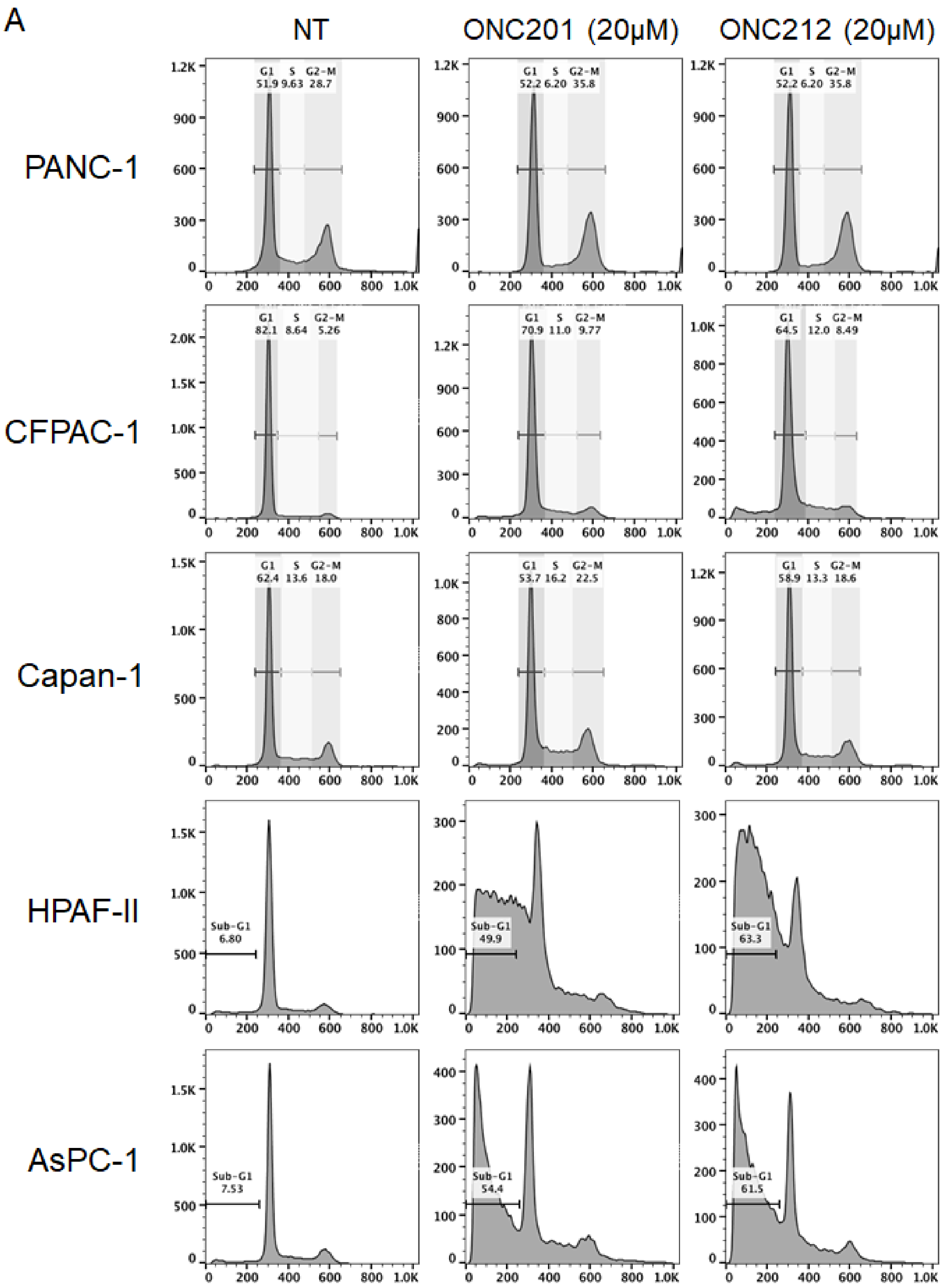
A
NT
ONC201 (20μM)
ONC212 (20μM)
PANC-1
G1 51.9 S 9.63 G2-M 28.7
G1 52.2 S 6.20 G2-M 35.8
G1 52.2 S 6.20 G2-M 35.8
CFPAC-1
G1 82.1 S 8.64 G2-M 5.26
G1 70.9 S 11.0 G2-M 9.77
G1 64.5 S 12.0 G2-M 8.49
Capan-1
G1 62.4 S 13.6 G2-M 18.0
G1 53.7 S 16.2 G2-M 22.5
G1 58.9 S 13.3 G2-M 18.6
HPAF-II
Sub-G1 6.80
Sub-G1 49.9
Sub-G1 63.3
AsPC-1
Sub-G1 7.53
Sub-G1 54.4
Sub-G1 61.5

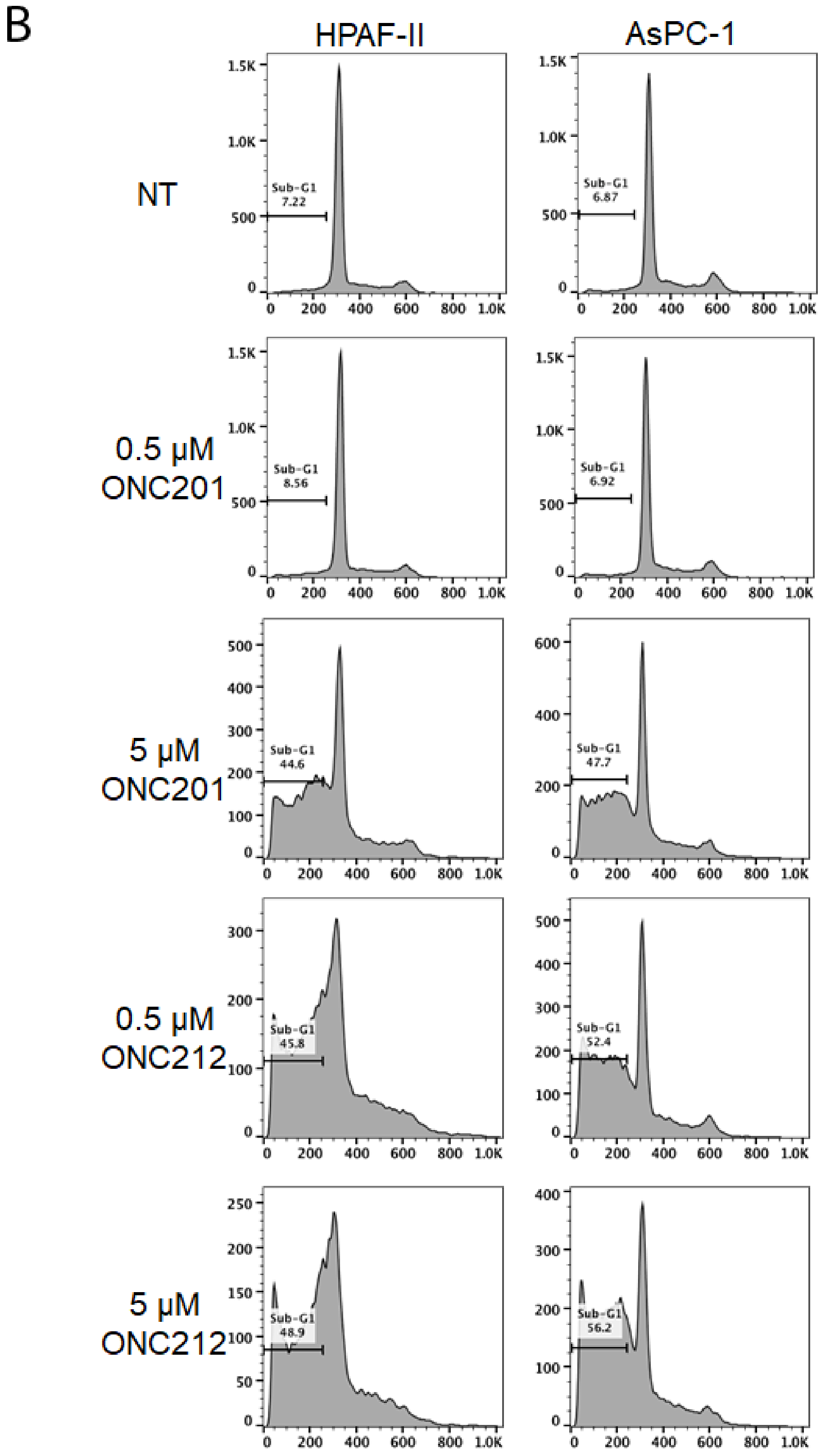

Supplementary Figure 2: (A) Histograms showing changes in different phases of the cell cycle in five human pancreatic cancer cell lines, 72 hours post-treatment**.** (**B**) Histograms indicating sub-G1 percentage in HPAF-II and AsPC-1 cell lines 72 hours post-treatment at the indicated doses of ONC201 or ONC212.

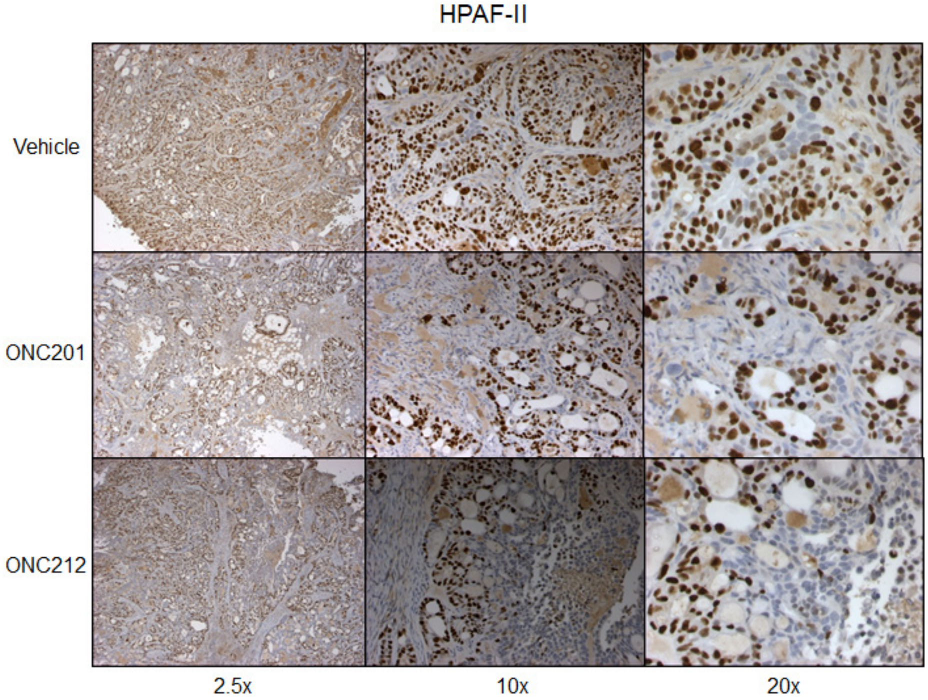

Supplementary Figure 3: Ki67 staining of HPAF-II tumor sections treated with either ONC201 or ONC212.

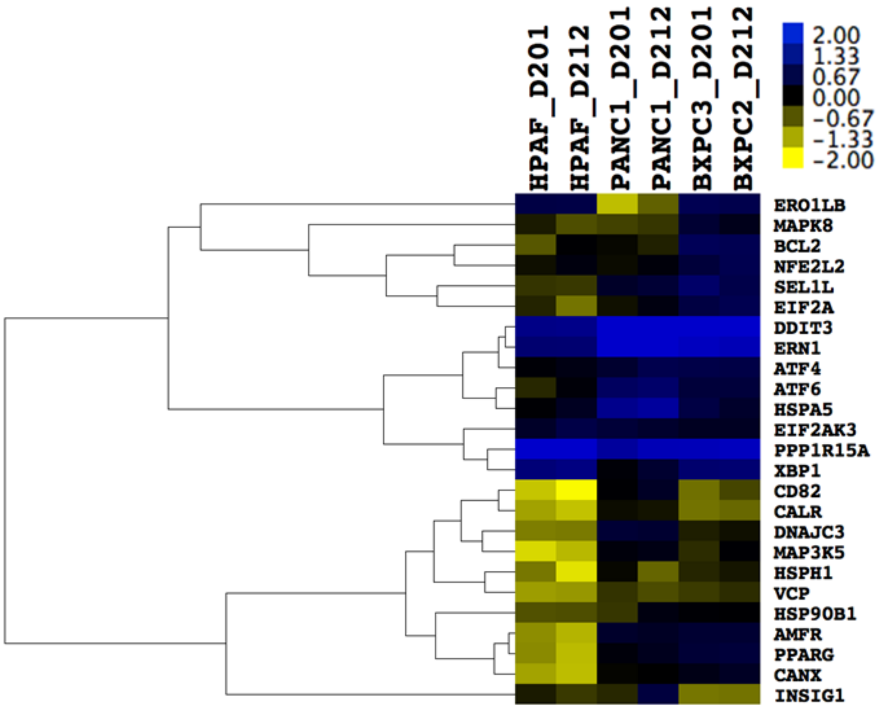

Supplementary Figure 4: Gene expression profiling by microarray analysis in HPAF-II, PANC-1 and BxPC3 cells 48 hours post treatment with 20 µM ONC201 or ONC212 in comparison to untreated cells. Representative heat map shows expression of UPR genes relative to control for each cell line.
